# The complete mitochondrial genome of ribbed gunnel *Dictyosoma burgeri* (van der Hoeven, 1855)

**DOI:** 10.1080/23802359.2020.1870882

**Published:** 2021-02-08

**Authors:** Yibang Wang, Hui Zhang, Weiwei Xian, Yue Zhang

**Affiliations:** a College of Marine Science and Biological Engineering, Qingdao University of Science and Technology, Qingdao, PR China; b CAS Key Laboratory of Marine Ecology and Environmental Sciences, Institute of Oceanology, Chinese Academy of Sciences, Qingdao, PR China; c University of Chinese Academy of Sciences, Beijing, PR China;; d Laboratory for Marine Ecology and Environmental Science, Qingdao National Laboratory for Marine Science and Technology, Qingdao, PR China; e Center for Ocean Mega-Science, Chinese Academy of Sciences, Qingdao, PR China

**Keywords:** Mitochondrial genome, *Dictyosoma burgeri*, ribbed gunnel, next-generation sequencing, phylogenetic

## Abstract

The complete mitochondrial genome of *Dictyosoma burgeri* collected from Yellow and Bohai Seas was determined by next-generation sequencing. The mitogenome is a circular molecule 16,513 bp in length, including the typical structure of 13 protein-coding genes, 2 ribosomal RNA genes, 22 transfer RNA genes, and a control region. The TAS, central CSB, and CSB were detected in the control region. The gene contents of the mitogenome are identical to those observed in most bony fishes.

*Dictyosoma burgeri* (van der Hoeven, 1855) is mainly distributed along the coasts of China, Japan and Korea (Yatsu et al. 1978). It has been described in detail for the seasonal gonadal development and histological observation in Jeju, Korea (Jin et al. 2007). Hwang et al. investigated the function of plasma and cortisol in female during the annual reproductive cycle (Hwang et al. 2012). However, ribbed gunnel has not received much attention in population genetic studies. Next generation sequencing (NGS) has increased the speed and throughput capacities of DNA sequencing, thus dramatically reducing the overall cost of sequencing (Zhang et al. 2011). The appearance of NGS has foster the characterization of mitochondrial genomes in various species (Li et al. 2019), which can be used to further understand population genetics and evolutionary history of species (Zhang and Xian 2016). In the present study, we extracted DNA from muscle tissue of ribbed gunnel collected from Bohai and Yellow Seas in the September 2019 and conducted appropriate NGS analysis to isolate mt DNA sequences.

In this study, whole Genome Shotgun (WGS) strategy was adopted to construct library. Paired-end (PE) Sequencing was performed using Next-Generation Sequencing (NGS) based on Illumina MiSeq Sequencing platform. A5-miseq V20150522 (Coil et al. 2015) and SPAdesv3.9.0 (Bankevich et al. 2012) were used to assemble high-quality second-generation sequencing data from bottom to construct contig and scaffold sequences. The splice complete mitochondrial genome sequence was uploaded to the MITOS Web server (http://mitos.bioinf.uni-leipzig.de/) for functional annotation (Bernt et al. 2013). Genetic code selects the setting as 05-Inverterbrate, and the rest Settings follow the default parameters set by MITOS.

The complete mitogenome of *Dictyosoma burgeri* was 16,513 bp in length (Genbank accession no. MT942974), within the range of other teleost mitogenomes. As in other vertebrates (Miya et al. 2001), it contained 13 protein-coding genes, 2 rRNA genes (12S rRNA and 16S rRNA), 22 tRNA genes, and a control region. Like other bony fishes, most mitochondrial genes of *D. burgeri* were encoded on the H-strand, with only ND6 and eight tRNA (Gln, Ala, Asn, Cys, Tyr, Ser, Glu, and Pro) genes encoded on the L-strand. Moreover, there were 3 overlapping reading frames in ND2 and ND5 genes, 4 in Cyt b gene. The ATPase 6 and ATPase 8 overlapped by 10 nucleotides, and ND4 and ND4L shared 7 nucleotides. ND5 and ND6 overlapped by 4 nucleotides on the opposite strand. ATG was the initiation codon of 12 out of the 13 protein coding genes (ND1, ND2, CO2, ATPase 8, ATPase 6, COIII, ND3, ND4L, ND4, ND5, ND6, and Cyt b), while the initiation codon of COI was GTG. TAA was the stop codon for 7 genes (ND2, COI, ATPase 8, ATPase 6, COIII, ND4L, and ND6), TAG was the stop codon for ND1, ND3 and ND5, the other genes had incomplete stop codons, either T (COII, ND4, and Cyt b), which were presumably completed as TAA by post-transcriptional polyadenylation (Ojala et al. 1981). The 12S and 16S ribosomal RNA genes of *D. burgeri* comprised 948 bp and 1668 bp, respectively. They were located between tRNA^Phe^ and tRNA^Leu^, and were separated by tRNA^Val^, as they were in other vertebrates (Gao et al. 2013). The 22 tRNA genes were interspersed in the genome and ranged in size from 66 to 74 bp and folded into cloverleaf secondary structures with normal base paring. The major noncoding region in *D. burgeri* was located between tRNA^Pro^ and tRNA^Phe^, and was determined to be 584 bp in length. The TAS, central CSB, and CSB were detected in the control region, which was similar to most bony fishes (Zhang et al. 2013). Phylogenetic analysis was accomplished by Mega X, using the complete sequence of *D. burgeri* and the other 10 closely related *Blenniidae* species. The Maximum Likelihood tree indicated that *D. burgeri* was sister species to *Pholis fangi* (Figure 1) and that the genus *Pholis* was not monophyletic.

**Figure 1.**
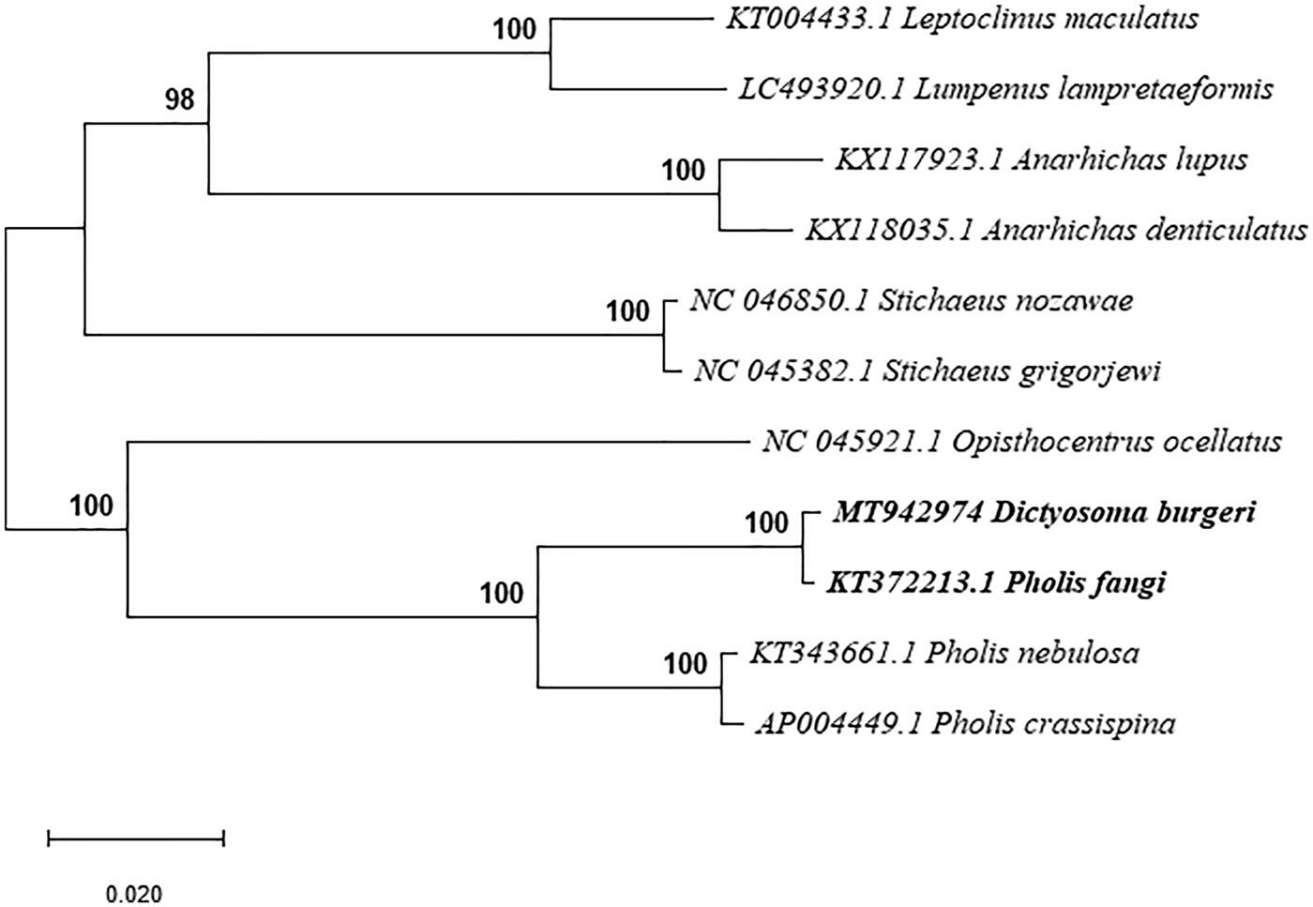
Phylogenetic relationship revealed by ML tree among 11 *Blenniidae* species.

## Data Availability

Mitogenome data supporting this study are openly available in GenBank at https://www.ncbi.nlm.nih.gov/nuccore/MT942974. Associated BioProject, SRA, and BioSample accession numbers are https://www.ncbi.nlm.nih.gov/bioproject/PRJNA678842, https://www.ncbi.nlm.nih.gov/sra/ SAMN16815269, respectively.
